# *Stomosis arachnophila* sp. n., a new kleptoparasitic species of freeloader flies (Diptera, Milichiidae)

**DOI:** 10.3897/zookeys.50.505

**Published:** 2010-06-30

**Authors:** Irina Brake, Michael von Tschirnhaus

**Affiliations:** 1 Department of Entomology, Natural History Museum, Cromwell Road, London SW7 5BD, United Kingdom; 2 Fakultät Biologie, Universität Bielefeld, Postfach 100 131, 33501 Bielefeld, Germany

**Keywords:** New species, Australia, kleptoparasitism

## Abstract

Stomosis arachnophila Brake **sp. n.** (Diptera, Milichiidae) is described from Western Australia. The species is kleptoparasitic on araneid spiders. The paper is an example for a new approach in cybertaxonomy which includes generation of manuscripts within a Virtual Research Environment (Scratchpads), semantic enhancement, parallel release of the publication on paper and online accompanied with registration of new taxa with ZooBank.

## Introduction

The milichiid genus Stomosis belongs to the subfamily Phyllomyzinae and includes five described species that occur in the New World and Australia. There are more than ten undescribed species just in Australia (Brake 2000) and IB has seen unidentified material possibly including undescribed species from the Afrotropical Region. Stomosis flava Sabrosky has been reared from puparia collected in tree cavities and some specimens were collected in a privy trap (Sabrosky 1958). The biology of the other described species is unknown.

During a field trip to southwestern Australia, MvT collected more than 200 Stomosis specimens belonging to at least three species, Stomosis vittata Malloch and two or three undescribed species of which one was used for a phylogenetic analysis of Milichiidae (Brake 2000). The aim of this paper is to describe the latter species to make its name available. A revision of the whole genus is badly needed but not feasible for the authors at this time.

## Material and methods

The terminology follows Brake (2009). We have tried to provide information on the color. However, the specimens have been in ethanol for several years and are partially cleared. It is therefore possible that brown parts were black originally. For the study of the terminalia, male abdomina were cleared in hot 10% aqueous potassium hydroxide solution then neutralized in acetic acid. Male abdomina were studied in glycerine and are preserved in euparal on a slide.

Specimens are deposited in the Australian National Insect Collection, Canberra (ANIC), Natural History Museum, London (BMNH), University of Bielefeld (UBI), National Museum of Natural History, Smithsonian Institution, Washington, DC (USNM), and Zoologische Staatssammlung München (ZSM).

This paper including the species description was prepared on the Milichiidae Online Scratchpad (www.milichiidae.info) as a test of a new method to publish nomenclatural acts described on taxonomic websites (Blagoderov et al. 2010). The paper has been semantically tagged and enhanced using the Pensoft Mark Up Tool (PMT) which is based on the US National Library of Medicine’s DTD (Document Type Definitions) TaxPub extension (Revision #123) (http://sourceforge.net/projects/taxpub). The final XML output of the paper has been archived in PubMedCentral, a PDF uploaded in the Biodiversity Heritage Library (BHL), and all revised species registered in ZooBank (Penev et al. 2010).

## Stomosis arachnophila

Brake sp. n.

urn:lsid:zoobank.org:act:267B9A8B-372C-45EC-BFE5-661AF13CABC8

Stomosis sp. 1. in Brake 2000: 13 (Pl. 1D, 7G, 8G, 10B, 12A+E, 15D+F) [morphology, phylogeny].

### Material examined.

#### Holotype, male:

Australia. Western Australia: SSW' Walpole, Coalmine Beach at northern bank of Nornalup Inlet, Caravan Park, also S' Walpole Inlet [34°59'S, 116°45'E , 9.III.1989, X649, M. von Tschirnhaus (UBI_IBC_3220248, ANIC). The holotype is in good condition and is glued to a paper triangle on a pin.

#### Paratypes (in ethanol if not otherwise stated):

same data as holotype, 10♂11♀ (UBI_IBC_3220246-7, ZSM, in ethanol, UBI_IBC_3220249-55, BMNH, ANIC, USNM glued to paper triangle). Australia. Western Australia: SSW' Walpole, Coalmine Beach at northern bank of Nornalup Inlet, Caravan Park, also S' Walpole Inlet [34°59'S, 116°45'E, 9.III.1989, X651, M. von Tschirnhaus, 5♂11♀ (UBI_IBC_3220119-20, BMNH); west coast, Canal Rocks, 4.5 km SSW' Yallingup, 12.4 km SW' Dunsborough [33°41'S, 115°00'E, sweep net, 21.II.1989, X631, M. von Tschirnhaus, 2♂ (UBI_IBC_3220111, USNM); west coast, Canal Rocks Beach Resort, 50 meter S' of beach, 3 km SSW' Yallingup, 10 km SW' Dunsborough [33°40'S, 115°01'E, sweep net, 23.II.1989, X632, M. von Tschirnhaus, 1♂4♀ (UBI_IBC_3220112-3, ZSM); western Stirling Range, Mondurup Peak, highest rock ridge, 800 to 817 m.a.s.l. [34°24'S, 117°47'E, sweep net, 26.III.1989, X676, M. von Tschirnhaus, 1♂2♀ (UBI_IBC_3220128-9, ZSM); south coast, Hopetoun, caravan park at sea site [33°57'S, 120°07'E, sweep net, 31.III.1989, X681, M. von Tschirnhaus, 1♂1♀ (UBI_IBC_3220130-1, ANIC).

#### Non type material:

same data as holotype, 35♂12♀1? (UBI_IBC_3220117-9, UBI, in ethanol), 3♂3♀ (UBI_IBC_3220256-61, BMNH, glued to paper triangle).

### Diagnostic description.

Differing from congeners in the combination of yellow anterior margin of frons, completely brown basoflagellomere, completely brown thorax and shape of male terminalia.

### Morphology.

#### Coloration and vestiture:

Head brown posteriorly, yellow anteriorly, slightly microtomentose except for frons. Frons usually brown on posterior 2/3 and yellow on anterior third of frons, but in a few specimens the frons is completely yellow except for a brown ocellar triangle and orbital plates; lunule yellow, face light brown; gena yellow except for posterior margin; antenna brown; palpus yellow with narrow brown tip and proboscis light brown. Thorax completely brown, slightly microtomentose except for shiny area posterior to base of fore coxa, wing and calypter hyaline, veins brown, halter white, legs black except for lighter ventral side of fore coxa and yellow narrow bases of all tibiae as well as distal tip of fore tibia, and all basitarsi. Abdomen with all tergites and sternites brown, slightly microtomentose except for anterior half of synsternite 7/8 and of epandrium.

#### Head:

Frons with 3 orbital setae, posterior seta lateroreclinate, the medial seta lateroclinate, anterior seta lateroproclinate, and 2 medioclinate frontal setae, postocellar setae medioclinate (cruciate). Arista about 2.5x as long as basoflagellomere width, pubescence on arista very short. Tip of ocellar triangle nearly reaching anterior third of frons. Gena height 0.13–0.15x eye height (Brake 2000, Pl. 1D).

#### Thorax:

2 dc, 1 prsc, 1 pprn, 1 prs, 1 sa, 1 pa, 1 keps setae, posterior prsc about a third as long as posterior dc, seta between posterior prsc and posterior dc absent.

#### Wing (Brake 2000, Pl. 10B):

with veins R4+5 and M parallel; M-ratio is 2.9; length: 1.7–2.1 mm.

#### Male abdomen:

**Figures 1–3.**
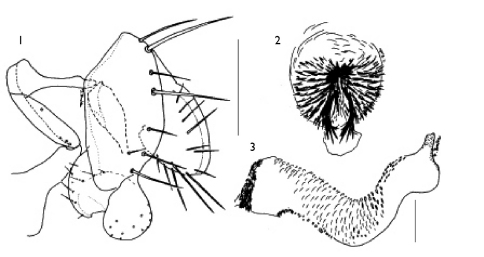
Stomosis arachnophila sp. n.: 1 male genitalia, lateral view 2 tip of distiphallus, apical view 3 distiphallus, lateral view. Scale: 0.1mm.

Sternite 5 divided into a narrow anterior and a narrow posterior part. Anterior part bent behind sternite 4 and covered with setulae, which may be part of a gland (Brake 2000, Pl. 12A). Male genitalia as in Figs. 1-3; distiphallus membranous and tubular, about as wide and 6–7x as long as epandrium length, with many setae at apex.

#### Size.

2.0–2.5 mm.

### Distribution.

Australia (Western Australia).

### Etymology.

The species name, arachnophila, is an adjective derived from the Greek arachnae = spider and philos = loving, referring to the kleptoparasitic behaviour of the species.

### Biology.

Field observations by M. von Tschirnhaus for sample X651: "16 milichiids are flying in front of a huge spider (Araneidae), which sits in the middle of her web. Finally the flies alight on a winged ant, which is caught in the web and is still alive, though hardly moves. There are several dead ants of the same species in the web as well as a number of silvery Therediidae. The female spider (deposited in UBI) stays for a long time in her place even when provoked. There are up to three milichiids on one prey, that has to be freshly dead. The two flies in sample X649, which were sitting on a Phonognatha (Araneidae) case (made from dead eucalyptus leaves) probably belong to the same species. 20 more cases were searched unsuccessfully. One milichiid alights on spider leg and runs up and down the leg dabbing, unheeded by the spider. One milichiid on ant with regurgitation droplet."

As described in the field notes, adults of the new species, Stomosis arachnophila, are kleptoparasitic on spiders.

### Discussion.

Kleptoparasitism, the stealing of food from another animal, is present in the stem species pattern of the Milichiidae and may also be present in the stem species pattern of its sister family, the Chloropidae (Brake 2000). Adults of some species in several milichiid genera feed by sucking on prey of spiders or predatory insects such as Reduviidae, Asilidae, Mantidae, or Odonata. Mostly they are attracted to predators feeding on stink bugs (Pentatomidae), squash bugs (Coreidae) or in the case of Desmometopa flies, on honey bees (Apidae) (Frost 1913; Robinson and Robinson 1977; Sivinski and Stowe 1980; Landau and Gaylor 1987). This is the first record of ants as prey.

In almost all cases it is only the female fly that is kleptoparasitic, possibly because kleptoparasitism provides the females with protein needed to produce eggs. In the present example, however, males and females were aspirated directly from the prey. It is possible that in this case the males are attracted to the spider's web in order to find mating partners.

Kleptoparasitism on spiders is known to be a habit of adults of some species in the genera Desmometopa, Milichiella, Neophyllomyza, Paramyia, Phyllomyza (Mik 1898; Biró 1899; Kertész 1899; Lundström 1906; Frost 1913; Kramer 1917; Robinson and Robinson 1977; Lopez 1984; Landau and Gaylor 1987; Sivinski and Stowe 1980; Nentwig 1985; Eisner et al. 1991; Stark and Schellhorn 2005). McMillan (1976) observed Desmometopa flies on Araneus and Nephila spiders (Araneidae) in Western Australia and these flies appeared to be acting as cleaners of the spiders, with the spiders spreading their wet and sticky chelicerae thus allowing the flies to feed actively all over the bases, fangs and mouth. McMillan also observed the flies to feed at the anal opening when the spiders defaecated. This behaviour appears to be more a case of commensalism, which is beneficial to both parties, than of kleptoparasitism.

## Supplementary Material

XML Treatment for Stomosis arachnophila

## Supplementary material

The Scratchpad version of this publication is available on the Milichiidae Online website at:

Photos of a male specimen are available at:

A distribution map is available on the Stomosis arachnophila species page at
